# CBX2 shapes chromatin accessibility promoting AML via p38 MAPK signaling pathway

**DOI:** 10.1186/s12943-022-01603-y

**Published:** 2022-06-09

**Authors:** Nunzio Del Gaudio, Antonella Di Costanzo, Ning Qing Liu, Lidio Conte, Carmela Dell’Aversana, Guglielmo Bove, Rosaria Benedetti, Liliana Montella, Fortunato Ciardiello, Vincenzo Carafa, Concetta Ambrosino, Valeria Tucci, Mariarosaria Conte, Joost H. A. Martens, Hendrik G. Stunnenberg, Angela Nebbioso, Lucia Altucci

**Affiliations:** 1 Department of Precision Medicine, University of Campania “Luigi Vanvitelli”, Vico L. De Crecchio 7, 80138 Naples, Italy; 2 Division of Gene Regulation, Netherlands Cancer Institute (NKI), Plesmanlaan 121, 1066 CX Amsterdam, The Netherlands; 3 Institute of Experimental Endocrinology and Oncology “Gaetano Salvatore” (IEOS), National Research Council (CNR), 80131 Naples, Italy; 4 Medical Oncology Complex Unit, “Santa Maria delle Grazie” Hospital, ASL Napoli 2 Nord, Naples, Italy; 5 Biogem Institute of Molecular Biology and Genetics, Ariano Irpino, Italy; 6 Department of Molecular Biology, Faculty of Science, Radboud Institute for Molecular Life Sciences, Radboud University, 6525 GA Nijmegen, The Netherlands; 7 Princess Maxima Centre for Pediatric Oncology, 3584 CS Utrecht, The Netherlands

**Keywords:** PcG, Leukemia, CBX2, Epigenetics, Chromatin readers, Cancer

## Abstract

**Background:**

The dynamic epigenome and proteins specialized in the interpretation of epigenetic marks critically contribute to leukemic pathogenesis but also offer alternative therapeutic avenues. Targeting newly discovered chromatin readers involved in leukemogenesis may thus provide new anticancer strategies. Accumulating evidence suggests that the PRC1 complex member CBX2 is overexpressed in solid tumors and promotes cancer cell survival. However, its role in leukemia is still unclear.

**Methods:**

We exploited reverse genetic approaches to investigate the role of CBX2 in human leukemic cell lines and ex vivo samples. We also analyzed phenotypic effects following CBX2 silencing using cellular and molecular assays and related functional mechanisms by ATAC-seq and RNA-seq. We then performed bioinformatic analysis of ChIP-seq data to explore the influence of histone modifications in CBX2-mediated open chromatin sites. Lastly, we used molecular assays to determine the contribution of CBX2-regulated pathways to leukemic phenotype.

**Results:**

We found CBX2 overexpressed in leukemia both in vitro and ex vivo samples compared to CD34^+^ cells. Decreased CBX2 RNA levels prompted a robust reduction in cell proliferation and induction of apoptosis. Similarly, sensitivity to CBX2 silencing was observed in primary acute myeloid leukemia samples. CBX2 suppression increased genome-wide chromatin accessibility followed by alteration of leukemic cell transcriptional programs, resulting in enrichment of cell death pathways and downregulation of survival genes. Intriguingly, CBX2 silencing induced epigenetic reprogramming at p38 MAPK-associated regulatory sites with consequent deregulation of gene expression.

**Conclusions:**

Our results identify CBX2 as a crucial player in leukemia progression and highlight a potential druggable CBX2-p38 MAPK network in AML.

**Supplementary Information:**

The online version contains supplementary material available at 10.1186/s12943-022-01603-y.

## Background

Epigenetic and genetic alterations drive cancer initiation and progression. Dysregulation of epigenetic players, the so-called readers, writers, and erasers, is a key feature of all human cancers, including leukemia [1, 2]. Acute myeloid leukemia (AML) is one of the most fatal forms of adult hematological malignancies. It is a heterogeneous and aggressive cancer type that displays an early block of hematopoietic cell differentiation and aberrant proliferation. AML is characterized by a remarkable genetic complexity due to different sets of cytogenetic alterations and somatic mutations [3]. In addition, mutations in epigenetic regulators such as DNMT3A, IDH1/2, TET2, EZH2, and ASXL1 appear to be crucial in the onset and progression of AML pathophysiology [1].

Mutations and/or deregulation of Polycomb group (PcG) proteins are described as epigenetic drivers in human cancers, including hematological malignancies [4]. Several studies demonstrated that deregulated PcG proteins affect cell proliferation, differentiation, and self-renewal, resulting in the altered trajectory of programmed cell fate [5]. PcG protein complexes are classified into two major multiprotein complexes: Polycomb repressive complex 1 (PRC1) and Polycomb repressive complex 2 (PRC2). PRC1 can be further divided into canonical (cPRC1) and non-canonical (ncPRC1) complexes and characterized by the presence or absence of chromobox-containing (CBX) protein subunits (CBX2, 4, 6, 7, 8) [6]. PRC1 and PRC2 catalyze histone H2A monoubiquitination at lysine 119 (H2AK119ub1) and the mono-, di-, and trimethylation of histone H3 at lysine 27 (H3K27me1/2/3). CBX subunits of cPRC1 are able to “read” (and bind to) H3K27me3 modifications, allowing recruitment of cPRC1 to PRC2 target genes and consequently resulting in chromatin compaction and transcriptional repression [7, 8]. CBX2 overexpression has been found implicated in promoting cancer cell proliferation [9–11].

Although an increasing number of studies report overexpression of CBX2 in different tumors, its oncogenic role remains poorly elucidated. We previously reported that the histone deacetylase inhibitor SAHA controls CBX2 stability in leukemias, and we unraveled the specific molecular mechanism regulating CBX2 protein stability [12].

Here, we propose CBX2 as a critical target in AML by showing that CBX2 is overexpressed and positively regulates AML cell proliferation and survival. CBX2 silencing arrests cell proliferation and promotes apoptotic cell death in both in vitro and ex vivo AML cells. Intriguingly, the silencing of CBX2 alters chromatin accessibility of regulatory sites associated with genes involved in cell proliferation and survival, leading to upregulation of apoptotic genes such as TNF-α and downregulation of genes involved in proliferative pathways such as KRAS and MAPK signaling.

Our findings demonstrate the functional role of CBX2 in AML development and progression, suggesting the potential therapeutic importance of CBX2 as a candidate target in AML.

## Materials and methods

### Cell culture

U937, K562, HL-60, and NB4 cells (DMSZ) were cultured in RPMI 1640 (Euroclone, Italy) supplemented with 10% fetal bovine serum (FBS) (Sigma-Aldrich, Italy), 2 mM L-glutamine (Euroclone), and antibiotics (100 U/mL penicillin, 100 μg/mL streptomycin, and 250 ng/mL amphotericin-B; all Euroclone). HEK293FT (DMSZ) cells were grown in Dulbecco’s modified Eagle’s medium (Euroclone) supplemented with 10% FBS, 100 U/mL penicillin/streptomycin (Euroclone), and 2 mM glutamine (Euroclone).

### Ex vivo cell culture

Leukemic blasts cells were obtained from the peripheral blood or bone marrow of leukemia patients and purified by Ficoll (Sigma-Aldrich). Cells were growth in RPMI 1640 (Euroclone) supplemented with 20% heat-inactivated FBS (Sigma-Aldrich), 1% glutamine (Euroclone), 1% penicillin/streptomycin (Euroclone), and 0.1% gentamicin (Euroclone). The experiments were approved by the University of Campania “Luigi Vanvitelli” Ethical Committee (Prot. number: 296). CD34^+^ cells were purchased from STEMCELL Technologies (#70002).

### Lentiviral production and infection

Lentivirus production and cell infection were performed as previously described [13].

### Western blot (WB) analysis

WB analysis was performed as previously reported [12]. Briefly, cells were lysed in RIPA buffer (1% Triton X-100, 0.1% SDS, 150 mM NaCl, 1 mM EDTA pH 8, 10 mM Tris-HCl pH 8) containing 1% protease inhibitor cocktail (Roche). Cell extract was centrifuged for 15 min at 4 °C and heated for 5 min at 95 °C; 30 μg of total protein extract was subjected to SDS-polyacrylamide gel electrophoresis, blotted on nitrocellulose membrane (Bio-Rad) and incubated ON with the specific antibodies. Protein expression was detected by ECL chemiluminescence method (Bio-Rad).

### RT-qPCR

RNA extraction was performed using TRIzol (Thermo Fisher Scientific) according to the supplier’s instructions. 500 ng RNA was reverse transcribed using the SuperScript VILO DNA Synthesis Kit (Thermo Fisher Scientific) as described in the manufacturer’s instructions. RT-qPCR was performed with a Bio-Rad iCycler iQ Real-Time PCR Detection System using iQ SYBR Green Supermix (Bio-Rad). Analysis was performed by ΔΔCt method.

### Antibodies, chemical reagents, and plasmids

Primary antibodies used for WB: anti-CBX2 (Bethyl Laboratories; #A302-524A), anti-ERK1/2 (Santa Cruz Biotechnology; #SC-94), anti-PARP (Abcam; #ab32138), anti-caspase-8 (Cell Signaling Technology; #9746S), anti-caspase-9 (Cell Signaling Technology; #9504S), anti-KRAS (Abcam; #ab180772), anti-pERK1/2 (Cell Signaling Technology; #9101S), anti-phospho-p38 (Abcam; #ab4822), anti-p38 (Abcam; #ab170099). Primary antibodies used for CUT&RUN: anti-CBX2 (Bethyl Laboratories; #A302-524A), anti-H3K27me3 (Thermo Fisher Scientific; # A15024) Antibodies were used according to the manufacturer’s instructions. Lentiviral shRNAs targeting CBX2 (TRCN0000020324, TRCN0000020326) and SCR control (TRC000035) plasmids were from the Sigma MISSION human shRNA library (Sigma-Aldrich).

### ATAC-seq

ATAC libraries of the shSCR and shCBX2 U937 cell lines were prepared using a reported protocol [14]. In brief, nuclei were isolated using a lysis buffer consisting of 10 mM Tris-HCl pH 7.5, 10 mM NaCl, 3 mM MgCl_2_, and 0.1% IGEPAL CA-630 detergent, and then tagmentated using 2 μl Tn5 transposase and 12.5 ul 2X TD buffer (Illumina; Nextera DNA Library Preparation Kit). The generated DNA fragments were amplified by two sequential 9-cycle PCR reactions, and after the first-round PCR reaction the libraries were selected for < 600 bp fragments using SPRI beads. The final PCR products were purified and quantified by KAPA Library Quantification Kits prior to sequencing. The libraries were sequenced with 43-cycle paired-end methods on the NextSeq 500 platform.

### RNA-seq

RNA of the shSCR and shCBX2 U937 cell lines was isolated using Trizol reagents (Life Technologies) according to the manufacturer’s instructions. Isolated RNA was subsequently subjected to on-column DNase treatment using the RNeasy Mini Kit (Qiagen), and ribosomal RNA was depleted from the resulting DNA-free RNA using the Ribo-Zero Gold Kit (Epicenter, Madison, WI, USA). Upon rRNA depletion, the RNA was fragmented and then 500 ng of fragmented RNA was reverse-transcribed using Random Hexane Primers (Invitrogen) and Super Script III Reverse Transcriptase (Invitrogen). RNA-seq libraries were prepared from the resulting cDNA samples following the standard KAPA Hyper Prep protocols and sequenced with 43-cycle paired-end methods on the NextSeq 500 platform.

### Cleavage under targets and release using nuclease (CUT&RUN)

CUT&RUN was used to profile chromatin binding sites of CBX2 and H3K27me. CUT&RUN was purchased from Cell Signaling Technology and used according to the manufacturer’s instructions. For each assay, nuclei were isolated from 2 × 10^5^ cells.

### ATAC-seq data processing

Raw sequencing data were mapped using BWA-MEM against the hg19 reference human genome. PCR duplicates and non-uniquely mapped reads (mapping quality score < 15) were removed for further data analysis. Peak calling was performed by MACS2 following default settings and at the q-value cutoff of 0.01. Only peaks called in two replicates of the same biological source were used for further analysis. GEO series accession numbers assigned were GSM5808307, GSM5808308, GSM5808309, GSM5808310.

### RNA-seq data processing

RNA-seq data were mapped using Bowtie mapper against CRCh37.72 reference human transcriptome, and values of expression were calculated using the MMSEQ package. Ensembl database (release 75) was used to annotate mapped transcripts. RNA-seq data were also mapped against the hg19 reference human genome using BWA-MEN and subsequently uploaded to the USCS genome browser for visualization. GSEA was performed against MSigDB version 5.2 gene sets. The gene sets were ranked using the “Diff_of_Classes” method. GEO series accession numbers assigned were GSM5808311 and GSM5808312.

### ChIP-seq data processing

ChIP-seq reads were aligned by BWA-ALN mapper on the hg19 reference human genome. PCR duplicates and reads marked with a mapping quality score < 15 were eliminated from the analysis. Peak calling was made by MASC2 using a *Q*-value cutoff of 0.01. Peaks were annotated using the Homer software.

ChIP-seq datasets used for this study are listed in the table below (Table 1):

**Table 1:** Table 1 ChIP-seq datasets

ChIP	Cell line and AML patients	GEO accession number
GFP-CBX2	K562	GSM1319305
GFP control	K562	GSM1319306
H3K27me3	K562	GSM1319307
H3K27me3	Kasumi-1	GSM1534446
H3K27me3	AML p1	GSM1612074
H3K27me3	AML p2	GSM1612061
H3K27me3	AML p3	GSM1612058
H3K27me3	AML p4	GSM1612056
H3K27me3	AML p5	GSM1612055
H3K27me3	AML p6	GSM1612053

### Annexin V assay and PI analysis

Phycoerythrin (PE) Annexin V-staining assay (BD Biosciences, USA) was carried out according to the supplier’s instructions. PI staining was performed as previously described [13]. Briefly, cells were collected, washed two times with PBS, and resuspended in PI buffer (0.2 μg/μL PI, PBS 1X). Subsequently, cells were analyzed on a FACS Celesta flow cytometer (BD Biosciences).

### Caspase activity assay

Caspase-8 activity was evaluated as previously reported [15]. Caspase8 Z-IETD and Caspase9 Z-LEHD inhibitors were purchased from R&D System and used at 50 μM.

### Cell counting Kit-8 assay (CCK8)

Cell counting Kit-8 assay was purchased from Elabscience and used according to the manufacturer’s instructions. CCK8 was used for assaying cell viability.

### Primers

*DUSP5* F: ACAGGCCAGCTTATGACCAG / R: CTGGTCATAAGCTGGCCTGT; *DUSP10* F: CTCAAGGCTGCGAATCTGAC / R: GTCAGATTCGCAGCCTTGAG; *CCND2* F: GATGCTGGAGGTCTGTGAGG / R: CCTCACAGACCTCCAGCATC; *CBX2* F: CAGAACCGGAAGAGAGGC / R: CCTCACAGACCTCCAGCATC; *GADPH* F: CACCATCTTCCAGGAGCG / R: CGCTCCTGGAAGATGGTG; *TNFSR14* F: TGTAGTCAAGGTGATCGTCTC / R: GAGACGATCACCTTGACTACA; *IL1B* F: GGAGAATGACCTGAGCACCT */* R: AGGTGCTCAGGTCATTCTCC; *MKI67* F AGGCAAAGAAGACCTGCT / R: AGCAGGTCTTCTTTGCCT. DUSP3 promoter (chr17:41,856,909-41,857,495) F: CTGTGCTGGGGTGGAGATAA / R: TTTCATGGCAGTAGGGGAGG; GADD45B promoter (chr19:24,751,20-24,771,20) F: CCCGTCACTGATCCCTCTTT / R: ATCAACTTGGCCGACTCGTA; MECOM promoter (chr3:169,381,269-169,381,814) F: CTCTCCCTCCCTCCTGTTTC / R: TGCTGGAGAGATAGCGAGTG; PRKCG promoter (chr19:54,385,396-54,386,651) F: CCCCTTTCTGCACTGACCTA / R: CCCCAGCTCTTACCTTGACA; GNG12 promoter (chr1:68,298,150-68,300,150) F: GAAGGGTTCAAGGTCGGGTA / R: CCCATCACCTTTAGCATCGC; FGF17 promoter (chr8:21,900,576-21,901,045) F: GAGGTTTGGAGGGGAGTGAG / R: TCAGAGGGAGGACAGAGACA; GATAD2B promoter (chr1:153,895,026-153,896,175) F: CTCGGCCATGCCAGTAGAAG / R: CCCCTCCTCCGTTTATGATTG; WBP1L promoter (chr10:104,502,944-104,504,093) F: GGGTTGGGACGAATTTTACACT / R: CGATCAAATATAAGTCCCGCCTTC; ATP5F1B promoter (chr12:57,039,306-57,040,455) F: CTGCAGTAGGCAGGTCCATTG / R: TTCAGATTAGCACCAGTTTTGACC; ALDH6A1 promoter (chr14:74,550,744-74,551,893) F: GTCCTGGGGATAAGTGATTGGTTA / R: CTCGGTAGGGAGTGTCCAAGTC.

### Functional enrichment analysis

GO terms were defined using MSigDB version 7.2 in GSEA software (http://software.broadinstitute.org/gsea/msigdb). Biological process, molecular function, and KEGG canonical pathway gene sets were analyzed and reported with FDR q-value < 0.05.

### Statistical analysis

Statistical analysis was assessed using unpaired *t*-test. *P* < 0.05 was considered significant. Experiments were performed in triplicate (unless otherwise stated).

## Results

### CBX2 is overexpressed in AML

**Fig. 1:**
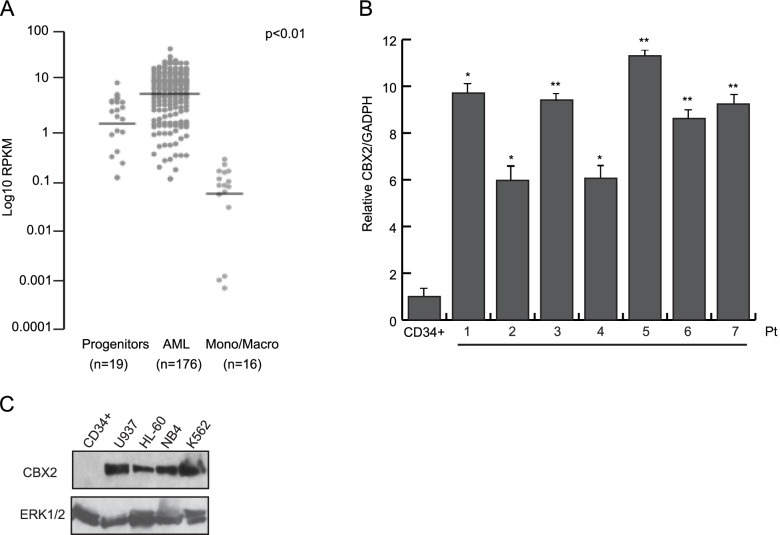
CBX2 is overexpressed in leukemia. **A** Dot plot representation of CBX2 expression in publicly available RNA-seq data from 176 primary human AML samples, 19 human hematopoietic progenitor samples, and 16 differentiated human monocyte and macrophage samples. RPKM values for each dataset were normalized to ACTB levels within each dataset and compared (*P* < 0.01, Welch’s *t*-test). **B** RT-qPCR analysis of CBX2 transcript in primary AML samples (pt) compared to CD34^+^ cells; error bars show SD of three biological replicates (*P < 0.05, **P ≤ 0.01). **C** WB analysis of CBX2 protein levels in a panel of leukemic cell lines and CD34^+^ cells; ERK1/2 was used as loading control

To explore the oncogenic potential of CBX2 in human leukemia, we analyzed CBX2 expression levels in 176 clinically annotated AML samples (TGCA) [16], 19 non-transformed human bone marrow progenitor samples, and 16 differentiated human monocyte and macrophage samples (blueprint cohort, https://www.blueprint-epigenome.eu) [17]. We found that the AML samples exhibited much higher CBX2 expression levels than hematopoietic progenitors and fully differentiated hematopoietic cells (Fig. 1A). Intriguingly, CBX2 expression was almost completely lost in differentiated monocytes and macrophages, likely suggesting an important role for CBX2 in counteracting terminal differentiation of mature blood cells (Fig. 1A). This observation was confirmed by interrogating the Bloodspot dataset (Supplementary Fig. 1B). Further, by using RT-qPCR we quantified the expression of CBX2 in several human primary AML ex vivo blasts. CBX2 RNA levels were significantly higher in all leukemic blasts than in CD34^+^ cells (STEMCELL Technologies #70002). (Fig. 1B). Additionally, WB analysis showed increased CBX2 protein expression in AML cell lines and in ex vivo AML samples compared to CD34^+^ myeloid progenitor cells (Fig. 1C and Supplementary Fig. 1A). Together, our data indicate that CBX2 overexpression critically contributes to the AML.

### CBX2 expression silencing leads to induction of apoptosis in leukemic cell lines

**Fig. 2:**
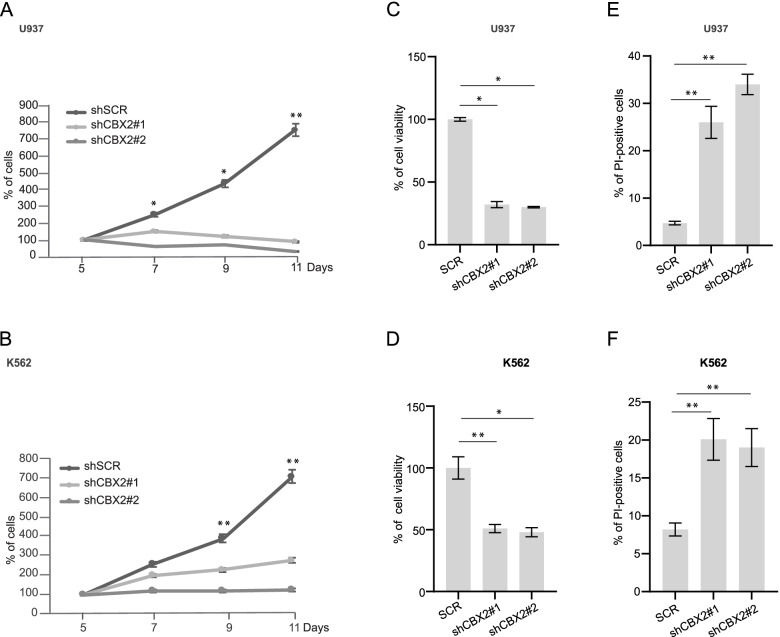
CBX2 silencing alters leukemic cell survival and induces cell death. **A** and **B** Proliferation assay with shCBX2#1, shCBX2#2, and shSCR in U937 (**A**) and K562 (**B**) cells; experiments were performed at 7 days of puromycin selection; error bars show SD of three biological replicates (*P < 0.05, **P ≤ 0.01). **C** and **D** CCK8 assay of CBX2 knock-down and shSCR-transduced U937 (**C**) and K562 (**D**) cells; experiments were performed at 7 days of puromycin selection; error bars show SD of three biological replicates (*P < 0.05, **P ≤ 0.01). **E** and **F** Percentage of PI-positive shCBX2- and shSCR-transduced U937 (**E**) and K562 (**F**) cells; experiments were performed at 7 days of puromycin selection; error bars show SD of three biological replicates (*P < 0.05, **P ≤ 0.01)

We further characterized the role of CBX2 in leukemic cell proliferation and survival at the molecular level. U937, K562, HL-60, and NB4 leukemia cells were transduced with lentiviruses expressing a non-targeting short hairpin RNA (shRNA) as control (shSCR) and two independent shRNAs targeting CBX2 gene (shCBX2) that display different knock-down efficacy (Supplementary Fig. 1C-F). Following puromycin selection, we performed cell growth rate analysis on shSCR and shCBX2 cells. CBX2 knock-down strongly reduced proliferation of AML cells, and growth inhibition was inversely correlated with CBX2 protein levels (Fig. 2A, B and Supplementary Fig. 1C-F, G, L). Additionally, CCK8 assay revealed that shCBX2 leads to a reduction of cell viability (Fig. 2C, D and Supplementary Fig. 1H, M). Interestingly, propidium iodide (PI) analysis showed an increase in cell death percentage in leukemic shCBX2- compared to shSCR-transduced cells (Fig. 2 E, F and Supplementary Fig. 1 I, N).

**Fig. 3:**
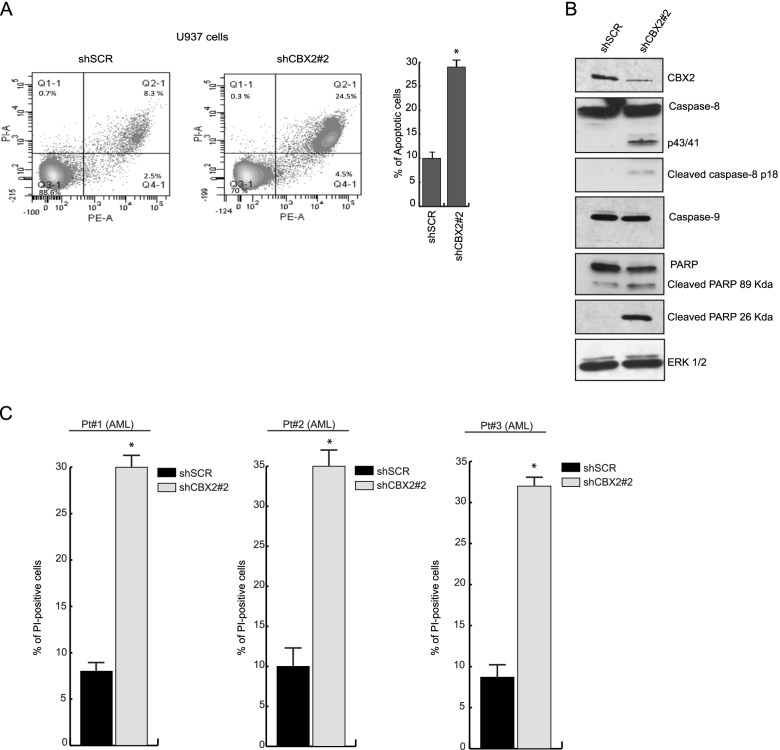
Knock-down of CBX2 induces apoptosis of AML cells. **A** Annexin V percentage of shCBX2- and shSCR-transduced cells; experiments were performed at 7 days of puromycin selection; error bars show SD of three biological replicates (*P < 0.05, **P ≤ 0.01). **B** WB analysis of the indicated proteins in shCBX2- and shSCR-transduced U937 cells at 7 days of puromycin selection. ERK1/2 was used as loading control; immunoblots were performed sequentially on the same membrane. **C** PI analysis of ex vivo AML blasts (from three different patients) knocked-down for CBX2. Experiments were performed at 3 days of puromycin selection; error bars show SD of two biological replicates

To better understand the molecular mechanisms underlying CBX2-mediated cell death, we measured the fraction of apoptotic annexin V-stained cells in CBX2-depleted U937 cells by flow cytometry analysis. shCBX2-transduced U937 cells displayed a higher percentage of apoptotic cells than shSCR cells (Fig. 3A). We then performed WB analysis to follow the key genes involved in induced programmed cell death of leukemic cells upon CBX2 knock-down. The results revealed cleavage and consequent activation of procaspase-8 (p43/41 kDa and p18 kDa fragments) and PARP (p27 kDa fragment) following CBX2 silencing. Proapoptotic events were closely correlated with CBX2 protein levels, indicating an on-target effect. Conversely, no procaspase-9 activation was observed upon CBX2 depletion (Fig. 3B).

To further investigate the shCBX2-induced cell death pathway, PI analysis was performed in shCBX2-transduced U937 cells treated with caspase-8 and caspase-9 inhibitors. As expected, inhibition of caspase-8, but not caspase-9, was able to rescue shCBX2-induced cell death (Supplementary Fig. 2B). Lastly, we also investigated the impact of CBX2 silencing in ex vivo AML blasts. Three ex vivo AML samples (Pt1, Pt2, and Pt3), were collected and transduced with the shCBX2 or scramble control (Supplementary Fig. 2C). PI analysis revealed an increased percentage of apoptotic cells in ex vivo AML blasts depleted for CBX2 compared to shSCR cells (Fig. 3C). Collectively, these data indicate that CBX2 depletion triggers cell cycle arrest and apoptosis in leukemic cell lines and ex vivo primary blasts, suggesting that CBX2 is critically required for AML cell survival.

### CBX2 depletion increases chromatin accessibility in AML cells

**Fig. 4:**
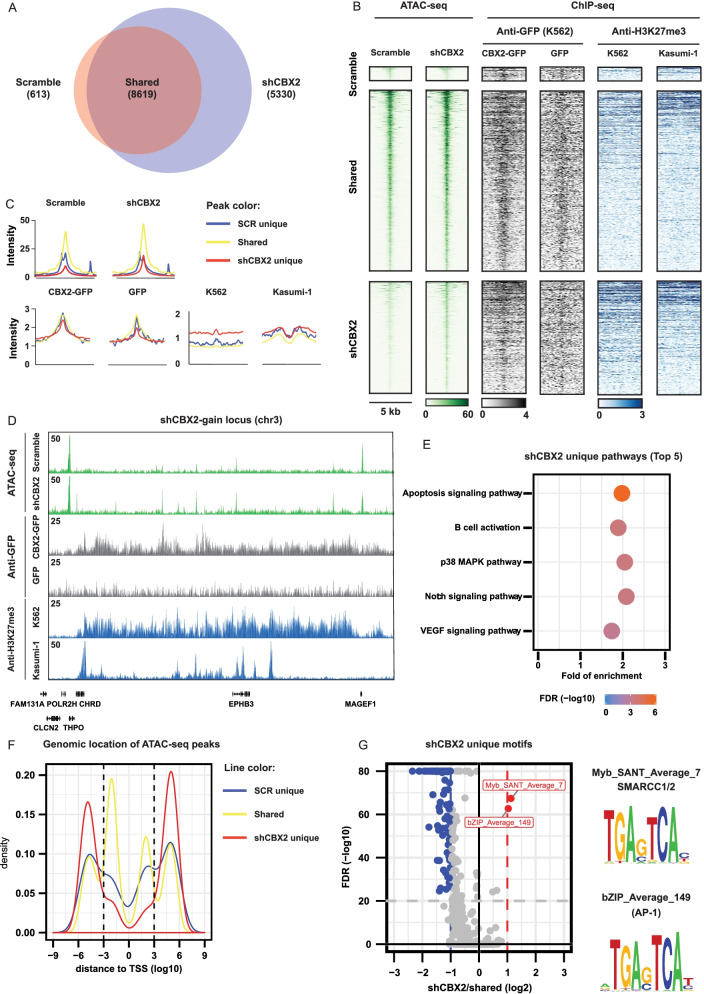
CBX2 silencing opens new chromatin regions in AML. **A** Venn diagram of ATAC-seq unique and shared peaks under shSCR and CBX2 knock-down conditions. **B** Heatmap analysis of ATAC-seq experiments showing shSCR and shCBX2 unique as well as shared regions in U937 cells; co-occurrence of GFP-CBX2, GFP and H3K27me3 occupancy at the ATAC-seq newly identified sites in the indicated cell lines. **C** Peak intensity quantification of shSCR, shCBX2, GFP negative control, GFP-CBX2 and H3K27me3 samples. **D** ATAC-seq examples of shCBX2 and shSCR samples together with GFP-CBX2, GFP and H3K27me3 occupancy at the indicated genomic locus. **E** Pathway analysis of shCBX2-associated unique regions; the top 5 significant enriched pathways are shown. **F** Genomic distance of shCBX2 unique, shSCR unique and shared ATAC-seq peaks from transcriptional start site. **G** Enrichment motif analysis of shCBX2 unique compared to shared sites

Chromatin accessibility has a functional role in gene regulation and thus in the establishment of transcriptional regulatory networks. As a subunit of PRC1, a major transcriptional repressor in eukaryotic cells, CBX2 may drive nucleosome compaction through its biochemically active chromatin compaction region [18]. We therefore hypothesized that CBX2 silencing may hamper the heterochromatic function of PRC1 and lead to the “decompaction” of some PRC1-repressed regions, consequently affecting expression of target genes. To follow changes in chromatin accessibility upon CBX2 depletion, we performed assay for transposase-accessible chromatin using sequencing (ATAC-seq). In U937 cells, CBX2 knock-down resulted in 5330 new open chromatin sites (Fig. 4A). An aggregate analysis identified a uniform increase in chromatin accessibility at these gained sites (Fig. 4B, C). For instance, the CHRD locus showed the clear formation of new open chromatin sites upon CBX2 knock-down (Fig. 4D). GREAT analysis indicated that the newly formed open chromatin sites are at (or close to) genes involved in important survival signaling transduction pathways, such as p38 MAPK and Notch pathways (Fig. 4E). Interestingly, we observed that the shCBX2-specific open chromatin sites are mainly enriched at more distal regulatory regions (Fig. 4F). Subsequently, to investigate the occupancy of CBX2 and H3K27me3 at shCBX2-induced new open chromatin sites, we analyzed CBX2 and H3K27me3 ChIP-seq datasets generated in K562 (GFP-CBX2 and H3K27me3) and Kasumi-1 (H3K27me3) cells. Interestingly, we observed a clear enrichment of CBX2 binding at shCBX2-induced open genomic sites, suggesting that these sites are regulated by CBX2 (Fig. 4 B, C). For example, the CHRD locus clearly showed the co-occurrence of CBX2 and H3K27me3 binding at the newly formed sites identified by ATAC-seq in shCBX2 (Fig. 4D). shCBX2-mediated open genomic regions lacking CBX2 and H3K27me3 binding were also found, suggesting that alternative epigenetic mechanisms might be involved in regulating these chromatin sites (Fig. 4B, C). The fact that H3K27me3 occupancy at CBX2 binding sites was weak, and often disposed in a broader manner with some cell-context specificities, indicates differences in potential direct vs indirect binding of CBX2 to the chromatin. Further, to identify potential transcription factors regulating these sites, we performed motif analysis and identified motifs for AP-1, a transcription factor of receptor tyrosine kinase signaling activated by the Ras/MAPK pathway, as significantly enriched motifs at shCBX2 unique sites (Fig. 4G). The binding motif for two SWI/SNF complex subunits (SMARCC1/2) was also enriched at these newly formed open chromatin sites (Fig. 4G). Remarkably, AP-1 cooperates with the SWI/SNF complex in response to growth factors and other extracellular stimuli that signal through Ras/MAPK for selection of cell-type specific enhancers possibly involved in several biological responses, including cell differentiation [19].

### CBX2 knock-down affects p38 MAPK pathway regulation

**Fig. 5:**
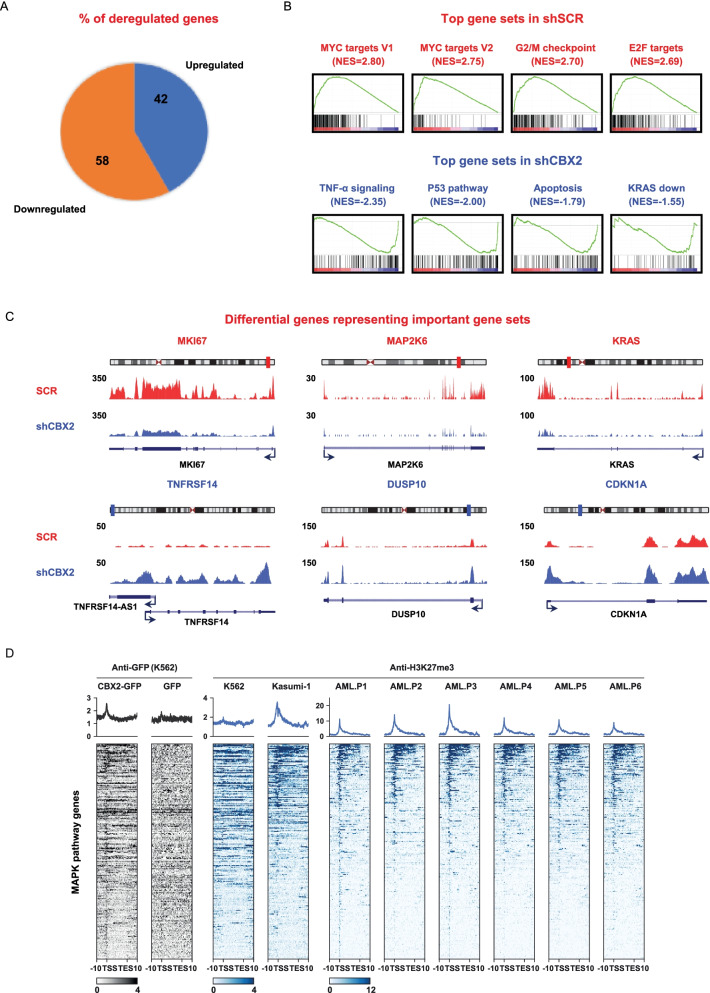
Silencing of CBX2 affects pathways tightly involved in cancer progression, including MAPK. **A** Pie chart showing upregulated and downregulated genes upon CBX2 knock-down. **B** GSEA analysis showing the top differential hallmark gene sets (nominal *P* < 0.05) related to shSCR and shCBX2 (NES = normalized enrichment score). **C** Simple track of representative differential genes with minimal 2-fold difference of key pathways upon CBX2 knock-down, including MAPK. **D** Heatmap analysis showing the co-occupancy of CBX2 and H3K27me3 at MAPK pathway regulatory regions in the indicated cell lines and in 6 different samples from AML patients

To investigate shCBX2-induced open chromatin-driven gene expression profiles, we performed whole transcriptome sequencing (RNA-seq) of CBX2 knock-down U937 cells compared to the scramble control. Data analysis identified a total of 1568 significantly (fold change ≥2) deregulated genes. Among these, 660 (42%) were upregulated and 908 (58%) downregulated in CBX2-depleted cells compared to shSCR (FDR ≤ 0.05) (Fig. 5A). Using gene set enrichment analysis (GSEA), we identified the top differential gene sets enriched or depleted in shCBX2 compared to non-targeting shRNA-transduced cells. CBX2-depleted samples revealed the downregulation of gene sets mainly related to proliferation, such as E2F and MYC targets, inflammation, and oxidative phosphorylation (Fig. 5B and Supplementary Fig. 2A), which is upregulated in several cancers including leukemia and lymphoma [20, 21]. Conversely, gene sets involved in apoptotic and antitumorigenic pathways, such as TNF-α and KRAS down-signaling, were enriched under knock-down conditions (Fig. 5B and Supplementary Fig. 2A).

**Fig. 6:**
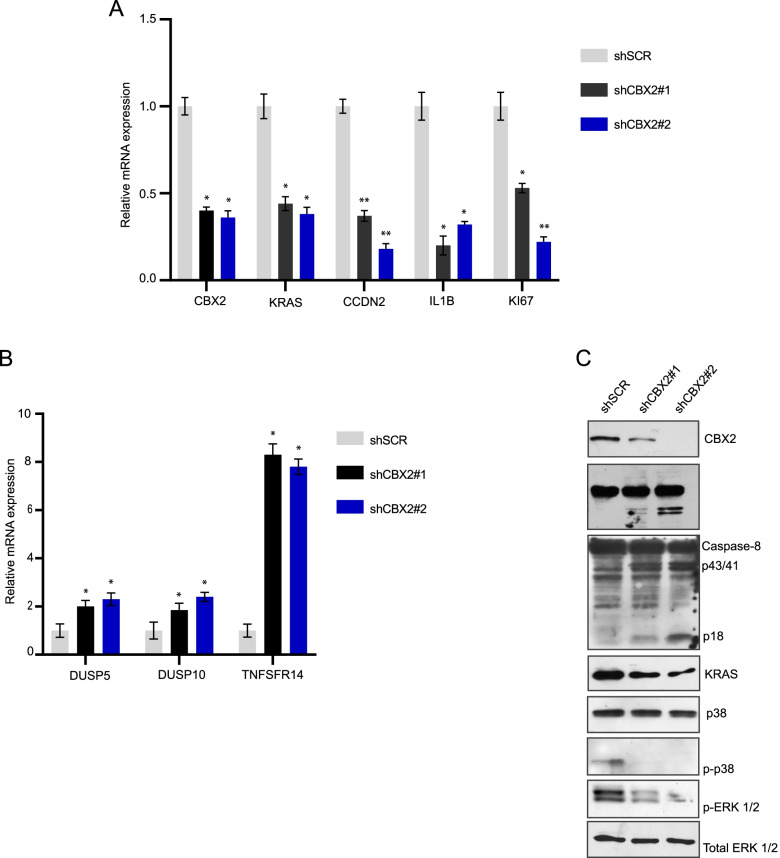
CBX2 depletion affects p38 MAPK pathway activation. **A** and **B** RT-qPCR analysis of indicated transcript in shCBX2#1, shCBX2#2, and shSCR U937 cells; error bars show SD of three biological replicates (*P < 0.05, **P ≤ 0.01). **C** WB analysis of indicated proteins in shCBX2#1, shCBX2#2, and shSCR U937 cells; ERK1/2 was used as loading control; immunoblots were performed sequentially on the same membrane

Notably, among the deregulated genes observed following CBX2 silencing we also found key genes functionally involved in MAPK pathway (Fig. 5C), whose activation was extensively shown to play a role in cancer progression [20, 22]. Specifically, in shCBX2 cells, we observed the upregulation of several MAPK phosphatases, including members of the dual-specificity phosphatase (DUSP) family such as DUSP5 and DUSP10, which preferably dephosphorylate ERK and p38, respectively, and the downregulation of KRAS and IL-1β, a key mediator of cell expansion and disease progression in AML following p38 MAPK activation [23] (Fig. 5C and Fig. 6 A, B).

In addition, we matched the ATAC-seq unique regions with differentially regulated genes (Supplementary Fig. 3A and B). Comparative analysis with differentially upregulated (UP_REG DR) genes in shCBX2 cells identified 223 shCBX2-induced open chromatin-associated genes whose transcripts were also upregulated (Supplementary Fig. 3A and Supplementary Table 1). Consistent with the RNA-seq results, Gene Ontology (GO) analysis of the 223 identified genes showed a significant enrichment of key biological processes, such as regulation of cell death, apoptosis process, cell proliferation, regulation of signaling pathway, and phosphorylation (Supplementary Fig. 3C). Notably, one of the most enriched and significant molecular function terms was kinase binding (Supplementary Fig. 4A). Among the 25 genes within this term, we also found negative regulators of the MAPK pathway, such as DUSP4, DUSP10, DUSP5, MAPK8IP3, and MAPK8IP2 (Supplementary Table 2). The potential CBX2-dependent regulation of MAPK signaling pathway was also verified by KEGG canonical pathway gene set enrichment analysis; 10 MAPK-associated genes (SPA1A, HSPA1B, TP53, TGFBR2, RASGRP3, DUSP10, DUSP4, DUSP5, MAPK8IP3, MAPK8IP2) were identified (Supplementary Fig. 4B). shCBX2-driven deregulated genes, including MAPK pathway-associated genes, were further validated by RT-qPCR (Fig. 6A and B).

### CBX2 impacts on p38 MAPK pathway and its depletion reduces leukemic survival

To gain insight into the molecular mechanism by which CBX2 depletion affects p38 MAPK pathway, we profiled CBX2 binding sites at p38 MAPK pathway genes by exploring a CBX2 ChIP-seq dataset (GFP-CBX2). We found that CBX2 binding occurs at p38 MAPK pathway gene regulatory sites (Fig. 5D). This result was further corroborated also in U937 cells by CUT&RUN following qPCR (Supplementary Fig. 2D). Further, we also explored the chromatin context at p38 MAPK pathway genes. By interrogating H3K27me3 ChIP-seq datasets in K562 and Kasumi-1 cells together with six additional different *primary* AML blast samples, we found that H3K27me3 co-occurs with CBX2 binding at p38 MAPK pathway gene regulatory regions. These results were again validated in U937 cells by CUT&RUN followed by qPCR (Fig. 5D and Supplementary Fig. 2E). Interestingly, we observed that the co-occupancy of CBX2 binding and H3K27me3 at MAPK pathway genes occurs mainly at promoter sites, indicating that CBX2 might be directly involved in the regulation of p38 MAPK pathway genes and that its deregulation may control a robust rearrangement of the p38 MAPK chromatin landscape via enrichment of H3K27me3, leading to alteration of p38 MAPK gene expression.

Activation of the MAPK pathway is widely considered crucial for AML cell growth and disease progression [24]. To further investigate the involvement of CBX2 in p38 MAPK pathway regulation, we analyzed ERK and p38 MAPK pathway activation status in CBX2 knock-down cells. Lower levels of p-p38, p-ERK and KRAS were observed in CBX2-depleted cells, indicating a CBX2-mediated sustainment of the pro-survival p38 MAPK pathway (Fig. 6C).

Additionally, GO enrichment of biological processes and molecular function, and KEGG pathway for 334 common genes identified by performing a supplementary comparative analysis between ATAC-seq unique regions and differentially downregulated (DOWN_REG DR) genes (Supplementary Fig. 3B and Supplementary Table 3) supported the specificity of shCBX2 in inducing chromatin remodeling and gene expression modulation in AML system (Supplementary Tables 4, 5 and 6).

Altogether, our data show that CBX2 depletion affects AML cell survival and deregulates key signal transduction pathways involved in AML maintenance and progression. Remarkably, our findings highlight the potential role of CBX2 in promoting p38 MAPK signal transduction pathways contributing to the AML phenotype.

## Discussion

The present study identifies CBX2 as a critical factor required for the AML and provides new insights into its functional role in contributing to leukemia cell onset and maintenance. We show that CBX2 is overexpressed in AML cells compared to CD34^+^ cells and to differentiated monocytes and macrophages, highlighting its possible function as a leukemia-driving oncogene. It is interesting to speculate that reduced CBX2 levels may be required for the physiological differentiation of myeloid lineage cells and that targeting CBX2 in leukemia might lead to differentiation. We further demonstrate that CBX2 is required for the growth of AML cells, consistent with the observed proliferative arrest and induction of caspase8-dependent apoptosis in CBX2 knock-down cells.

Depletion of CBX2 leads to increased genome-wide chromatin accessibility and altered leukemic cell transcriptional programs, inducing enrichment of cell death pathways and downregulation of genes regulating survival. Surprisingly, we observed that CBX2 silencing leads to epigenetic reprogramming of chromatin accessibility at regulatory sites associated with p38 MAPK signaling, which in turn alters the expression of related genes. Motifs of AP-1, a well-known transcription regulator of MAPK pathway-related genes, were in fact identified in CBX2 knock-down-specific open chromatin sites. Additionally, downregulation of positive regulators of p38 MAPK signaling, such as KRAS, and upregulation of negative p38 MAPK pathway regulators, such as DUSPs, were also identified in CBX2-depleted cells. Additionally, profiling of CBX2 binding sites showed that CBX2 occupancy occurs at p38 MAPK pathway gene promoters. It is tempting to speculate a direct regulatory role for CBX2 in sustaining activation of p38 MAPK signaling in AML. Similarly, silencing of CBX2 negatively affects RAS as well as p-ERK protein levels, revealing a new druggable CBX2-p38 MAPK axis required for AML progression.

Notably, shCBX2-mediated open chromatin sites were identified to be CBX2 binding regions by CBX2-ChIP-seq and display H3K27me3 occupancy. This histone mark maintains the chromatin state in silent mode and is recognized by CBX proteins, allowing the binding of PRC1 to chromatin. Hence, the presence of both CBX2 and H3K27me3 at CBX2-induced open genomic regions suggests that potential crosstalk mechanisms between PRC1 and PRC2 may occur influencing the chromatin state. However, since some CBX2-regulated open regions also show an H3K27me3-independent regulation, it is possible that other CBX2-related epigenetic mechanisms might be involved in their regulation. Indeed, some CBXs were recently reported to bind DNA and recruit PRC1 to DNA regulatory regions [25], warranting future investigations for alternative mechanisms by which PRC1 (and possibly PRC2) might be able to regulate the silent state of chromatin.

Although, the negative regulation of KRAS occurs at RNA level (Fig. 6A), analysis of publicly available CBX2 ChIP-seq datasets shows that the KRAS gene does not seem to be a primary CBX2 target (data not shown). We therefore speculate that CBX2 silencing may cause epigenetic changes resulting in the upregulation of transcription factors involved in the negative regulation of KRAS transcription. In support of this hypothesis, we found that genes involved in small GTPase mediated signal transduction, such as RASGRP2, are differentially expressed in RNA-seq data of shCBX2 cells and may be responsible for KRAS downregulation.

MAPK is a fundamental interconnected signaling cascade complex involved in the progression of several tumors. It consists of a large number of kinases that obtain gain-of-function mutations during oncogenic transformation [26]. Inhibiting CBX2 may therefore represent a possible strategy to target not only leukemia but also MAPK-driven tumorigenesis. In addition, the MAPK pathway is implicated in activating compensatory regulators in response to MAPK inhibitors and driving anticancer drug resistance [26]. By demonstrating the role of CBX2 in supporting the p38 MAPK signaling pathway, this study paves the way for exploring an alternative epigenetic approach to address MAPK-driven drug resistance by targeting PRC1 activity. Our findings also lay the groundwork for future investigations into CBX2-dependent p38 MAPK signaling in multiple cancer contexts.

Although in different cancer settings, previous studies [27–29] reported inhibition of p38 MAPK-triggered apoptosis via activation of caspase-8 and/or caspase-9. Here, we observed activation of caspase-8 but not caspase 9 upon depletion of CBX2 in AML cells, suggesting that, at least in this context, the extrinsic apoptotic pathway is more sensitive to MAPK inhibition. Additionally, since many Bcl-2 family proteins are implicated in both pro- and anti-apoptotic processes, and are under the control of p38 MAPK pathway, activation and/or inhibition of different apoptotic factors might contribute to caspase-8, but not caspase-9 activation. Further, RNA-seq analysis also revealed the overexpression of TNF-α signaling pathway, which is known to induce the apoptosis process via activation of caspase-8 [30]. Therefore, activation of this pathway might also contribute to caspase-8 induction, possibly explaining why caspase-8 but not caspase-9 activation was observed following CBX2 silencing.

## Conclusions

Our findings identify CBX2 as a critical target required for sustaining the leukemic phenotype. Mechanistically, CBX2 suppression leads to increased genome-wide chromatin accessibility that consequently alters AML transcriptional programs, inducing cell death pathways and silencing genes positively regulating cell survival. Further, our results highlight that CBX2 binds p38 MAPK pathway regulatory regions and that its knock-down reprograms chromatin regions at p38 MAPK-associated regulatory sites and alters the expression of genes belonging to p38 MAPK signaling pathway as well as its activity, thus affecting leukemic cell survival. Taken together, our data identify a novel druggable CBX2-p38 MAPK network, highlighting its potential as a new therapeutic strategy in leukemia and, potentially, in other p38 MAPK-driven cancers.

## Supplementary Information

**Additional file 1: Supplementary Figure 1.** CBX2 silencing impairs leukemic cell survival and induces cell death. **A** Western blot analysis of CBX2 protein levels in *ex vivo* leukemic blasts and CD34^+^ cells. Tubulin was used as loading control. Quantitative analysis (densitometry) of WB is shown as fold change value. **B** Bar plot representation of CBX2 expression in TGCA, blueprint and bloodspot datasets (*P* ≤ 0.01). **C-F** WB analysis showing silencing of CBX2 following shCBX2#1 and shCBX2#2 transduction in U937 (**C**), K562 (**D**), HL-60 (**E**), and NB4 (**F**) cells. ERK1/2 was used as loading control. **G** and **L** Proliferation assay with shCBX2(1), shCBX2(2), and shSCR in HL60 (**G**) and NB4 (**L**) cells; experiments were performed at 7 days of puromycin selection; error bars show SD of three biological replicates (*P < 0.05, **P ≤ 0.01). **H** and **M** CCK8 analysis of shCBX2- and shSCR-transduced HL60 (**H**) and NB4 (**M**) cells; experiments were performed at 7 days of puromycin selection; error bars show SD of three biological replicates (*P < 0.05, **P ≤ 0.01). **I** and **N** Percentage of PI-positive shCBX2- and shSCR-transduced HL60 (**I**) and NB4 (**N**) cells; experiments were performed at 7 days of puromycin selection; error bars show SD of three biological replicates (*P < 0.05, **P ≤ 0.01). **Supplementary Figure 2.** shCBX2 affects key pathways in AML. **A** GSEA analysis showing the top differential hallmark gene sets (nominal *P* < 0.05) associated with shSCR and shCBX2 cells (NES = normalized enrichment score). **B** Caspase activity assay of shCBX2- and shSCR-transduced U937 cells untreated or treated with 50 μM of caspase-8 or caspase-9 inhibitors; error bars show SD of three biological replicates. **C** RT-qPCR analysis showing CBX2 expression in three shCBX2#2- and shSCR-transduced primary AML samples (pt#1, pt#2 and pt#3). Experiments were performed at 3 days of puromycin selection; error bars show SD of two biological replicates (*P < 0.05, **P ≤ 0.01). **D** and **E** CUT&RUN-qPCR analysis showing enrichment of CBX2 (**D**) and H3K27me3 (**E**) at regulatory regions of indicated positive targets belonging to MAPK pathway. No CBX2 enrichment was observed at negative regions. Results were normalized for the input and expressed as fold change compared to rabbit IGG. Error bars indicate SD of three biological replicates (*P <0.05, **P ≤0.01). **Supplementary Figure 3.** ATAC-seq and transcriptional expression integration analysis. **A** Venn diagram showing the 223 common genes from comparison between unique regions by ATAC-seq and differentially upregulated (UP_REG DR) genes by RNA-seq in U937 shCBX2 cells. **B** Venn diagram showing the 334 common genes from comparison between unique regions by ATAC-seq and differentially downregulated (DOWN_REG DR) genes by RNA-seq in U937 shCBX2 cells. **C** GO enrichment analysis of the 223 common genes. GO biological processes are reported, indicating the number of genes enriched and statistical significance with FDR q-value. **Supplementary Figure 4.** GO molecular function and KEGG pathway enrichment analysis of 223 common genes. **A** GO molecular function and KEGG canonical pathway gene sets of the 223 common genes. **B** Number of genes enriched and statistical significance with FDR q-value are reported.

**Additional file 2: Supplementary Table 1.** List of 223 common genes from comparison between unique regions by ATAC-seq and differentially upregulated (UP_REG DR) genes by RNA-seq in U937 shCBX2 cells. **Supplementary Table 2.** List of 25 genes included in the GO molecular function kinase binding (FDR q-value = 1.95 e-9). **Supplementary Table 3.** List of 334 common genes from comparison between unique regions by ATAC-seq and differentially downregulated (DOWN_REG DR) genes by RNA-seq in U937 shCBX2 cells. **Supplementary Table 4.** GO biological processes of 334 common genes from comparison between unique regions by ATAC-seq and the differentially downregulated (DOWN_REG DR) genes by RNA-seq in U937 shCBX2 cells. **Supplementary Table 5.** GO Molecular Function of 334 common genes from comparison between unique regions by ATAC-seq and differentially downregulated (DOWN_REG DR) genes by RNA-seq in U937 shCBX2 cells. **Supplementary Table 6.** GO KEGG pathways of 334 common genes from comparison between unique regions by ATAC-seq and differentially downregulated (DOWN_REG DR) genes by RNA-seq in U937 shCBX2 cells.

## Data Availability

The data and materials of this study are available from the corresponding author upon request. For ATAC-seq data the GEO series accession numbers are GSM5808307, GSM5808308, GSM5808309, GSM5808310. For RNA-seq data the GEO series accession numbers are GSM5808311 and GSM5808312.

## Abbreviations

AML: Acute myeloid leukemia
PcG: Polycomb group
PRC1: Polycomb repressive complex 1
PRC2: Polycomb repressive complex 2
CBX: Chrombox-containing protein
WB: Western blot
PE: Phycoerythrin
CCK8: Cell counting Kit-8 assay
shRNA: Short hairpin RNA
PI: Propidium iodide
CUT&RUN: Cleavage Under Targets and Release Using Nuclease
ATAC-seq: Assay for transposase-accessible chromatin using sequencing
RNA-seq: Whole transcriptome sequencing
GSEA: Gene set enrichment analysis
DUSP: Dual-specificity phosphatase
GO: Gene ontology
UP_REG DR: Differentially upregulated genes
DOWN_REG DR: Differentially downregulated genes

## References

[1] Abdel-Wahab O, Levine RL (2013). Mutations in epigenetic modifiers in the pathogenesis and therapy of acute myeloid leukemia. Blood.

[2] Di Costanzo A, Del Gaudio N, Migliaccio A, Altucci L (2014). Epigenetic drugs against cancer: an evolving landscape. Arch Toxicol.

[3] Kidwell CS, Jahan R, Gornbein J, Alger JR, Nenov V, Ajani Z, Feng L, Meyer BC, Olson S, Schwamm LH (2013). A trial of imaging selection and endovascular treatment for ischemic stroke. N Engl J Med.

[4] Kaito S, Iwama A (2020). Pathogenic impacts of dysregulated Polycomb repressive complex function in hematological malignancies. Int J Mol Sci.

[5] Sparmann A, van Lohuizen M (2006). Polycomb silencers control cell fate, development and cancer. Nat Rev Cancer.

[6] Gao Z, Zhang J, Bonasio R, Strino F, Sawai A, Parisi F, Kluger Y, Reinberg D (2012). PCGF homologs, CBX proteins, and RYBP define functionally distinct PRC1 family complexes. Mol Cell.

[7] Kloet SL, Makowski MM, Baymaz HI, van Voorthuijsen L, Karemaker ID, Santanach A, Jansen P, Di Croce L, Vermeulen M (2016). The dynamic interactome and genomic targets of Polycomb complexes during stem-cell differentiation. Nat Struct Mol Biol.

[8] van den Boom V, Maat H, Geugien M, Rodriguez Lopez A, Sotoca AM, Jaques J, Brouwers-Vos AZ, Fusetti F, Groen RW, Yuan H (2016). Non-canonical PRC1.1 targets active genes independent of H3K27me3 and is essential for Leukemogenesis. Cell Rep.

[9] Chen WY, Zhang XY, Liu T, Liu Y, Zhao YS, Pang D (2017). Chromobox homolog 2 protein: a novel biomarker for predicting prognosis and Taxol sensitivity in patients with breast cancer. Oncol Lett.

[10] Clermont PL, Crea F, Chiang YT, Lin D, Zhang A, Wang JZ, Parolia A, Wu R, Xue H, Wang Y (2016). Identification of the epigenetic reader CBX2 as a potential drug target in advanced prostate cancer. Clin Epigenetics.

[11] Mao J, Tian Y, Wang C, Jiang K, Li R, Yao Y, Zhang R, Sun D, Liang R, Gao Z (2019). CBX2 regulates proliferation and apoptosis via the phosphorylation of YAP in hepatocellular carcinoma. J Cancer.

[12] Di Costanzo A, Del Gaudio N, Conte L, Dell'Aversana C, Vermeulen M, de The H, Migliaccio A, Nebbioso A, Altucci L (2018). The HDAC inhibitor SAHA regulates CBX2 stability via a SUMO-triggered ubiquitin-mediated pathway in leukemia. Oncogene.

[13] Del Gaudio N, Di Costanzo A, Liu NQ, Conte L, Migliaccio A, Vermeulen M, Martens JHA, Stunnenberg HG, Nebbioso A, Altucci L (2019). BRD9 binds cell type-specific chromatin regions regulating leukemic cell survival via STAT5 inhibition. Cell Death Dis.

[14] Liu NQ, Ter Huurne M, Nguyen LN, Peng T, Wang SY, Studd JB, Joshi O, Ongen H, Bramsen JB, Yan J (2017). The non-coding variant rs1800734 enhances DCLK3 expression through long-range interaction and promotes colorectal cancer progression. Nat Commun.

[15] Dell'Aversana C, Giorgio C, D'Amato L, Lania G, Matarese F, Saeed S, Di Costanzo A, Belsito Petrizzi V, Ingenito C, Martens JHA (2018). miR-194-5p/BCLAF1 deregulation in AML tumorigenesis. Leukemia.

[16] Ley TJ, Miller C, Ding L, Raphael BJ, Mungall AJ, Robertson A, Hoadley K, Triche TJ, Laird PW, Cancer Genome Atlas Research N (2013). Genomic and epigenomic landscapes of adult de novo acute myeloid leukemia. N Engl J Med.

[17] Chen L, Kostadima M, Martens JHA, Canu G, Garcia SP, Turro E, Downes K, Macaulay IC, Bielczyk-Maczynska E, Coe S (2014). Transcriptional diversity during lineage commitment of human blood progenitors. Science.

[18] Lau MS, Schwartz MG, Kundu S, Savol AJ, Wang PI, Marr SK, Grau DJ, Schorderet P, Sadreyev RI, Tabin CJ, Kingston RE (2017). Mutation of a nucleosome compaction region disrupts Polycomb-mediated axial patterning. Science.

[19] Vierbuchen T, Ling E, Cowley CJ, Couch CH, Wang X, Harmin DA, Roberts CWM, Greenberg ME (2017). AP-1 transcription factors and the BAF complex mediate signal-dependent enhancer selection. Mol Cell.

[20] Wagner EF, Nebreda AR (2009). Signal integration by JNK and p38 MAPK pathways in cancer development. Nat Rev Cancer.

[21] Ashton TM, McKenna WG, Kunz-Schughart LA, Higgins GS (2018). Oxidative phosphorylation as an emerging target in Cancer therapy. Clin Cancer Res.

[22] Santarpia L, Lippman SM, El-Naggar AK (2012). Targeting the MAPK-RAS-RAF signaling pathway in cancer therapy. Expert Opin Ther Targets.

[23] Carey A, DKt E, Eide CA, Newell L, Traer E, Medeiros BC, Pollyea DA, Deininger MW, Collins RH, Tyner JW (2017). Identification of Interleukin-1 by functional screening as a key mediator of cellular expansion and disease progression in acute myeloid leukemia. Cell Rep.

[24] Johnson DB, Smalley KS, Sosman JA (2014). Molecular pathways: targeting NRAS in melanoma and acute myelogenous leukemia. Clin Cancer Res.

[25] Piunti A, Shilatifard A (2021). The roles of Polycomb repressive complexes in mammalian development and cancer. Nat Rev Mol Cell Biol.

[26] Braicu C, Buse M, Busuioc C, Drula R, Gulei D, Raduly L, Rusu A, Irimie A, Atanasov AG, Slaby O (2019). A comprehensive review on MAPK: a promising therapeutic target in Cancer. Cancers (Basel).

[27] Yong HY, Koh MS, Moon A (2009). The p38 MAPK inhibitors for the treatment of inflammatory diseases and cancer. Expert Opin Investig Drugs.

[28] Papademetrio DL, Lompardia SL, Simunovich T, Costantino S, Mihalez CY, Cavaliere V, Alvarez E (2016). Inhibition of survival pathways MAPK and NF-kB triggers apoptosis in pancreatic ductal adenocarcinoma cells via suppression of autophagy. Target Oncol.

[29] Alvarado-Kristensson M, Melander F, Leandersson K, Ronnstrand L, Wernstedt C, Andersson T (2004). p38-MAPK signals survival by phosphorylation of caspase-8 and caspase-3 in human neutrophils. J Exp Med.

[30] Wang L, Du F, Wang X (2008). TNF-alpha induces two distinct caspase-8 activation pathways. Cell.

