# Supplementary material for: CBX2 shapes chromatin accessibility promoting AML via p38 MAPK signaling pathway
Source: Mol Cancer. 2022 Jun 9;21:125. doi: 10.1186/s12943-022-01603-y (PMC9178829; doi:10.1186/s12943-022-01603-y)

Supplementary figure 1

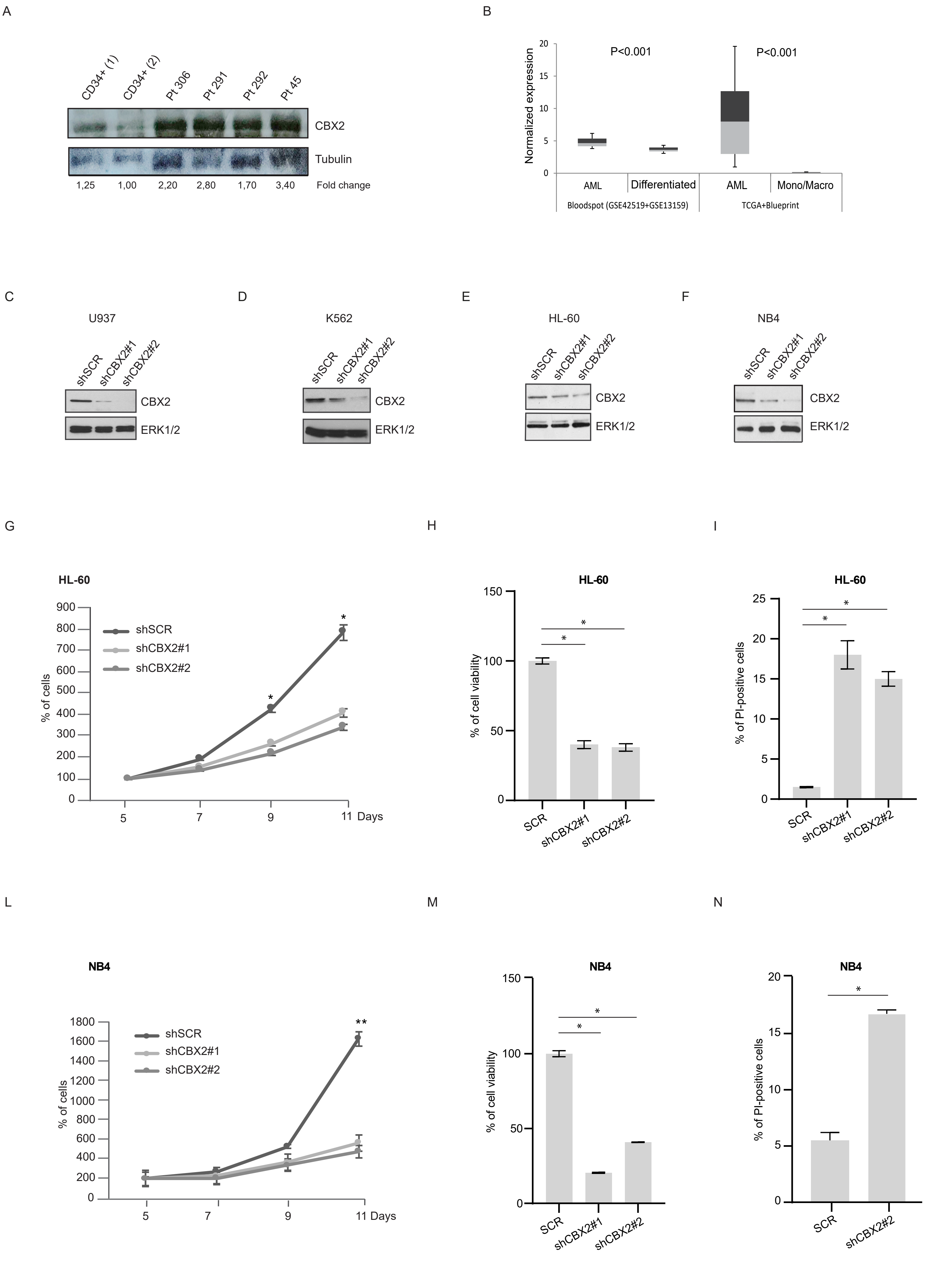

Supplementary figure 2

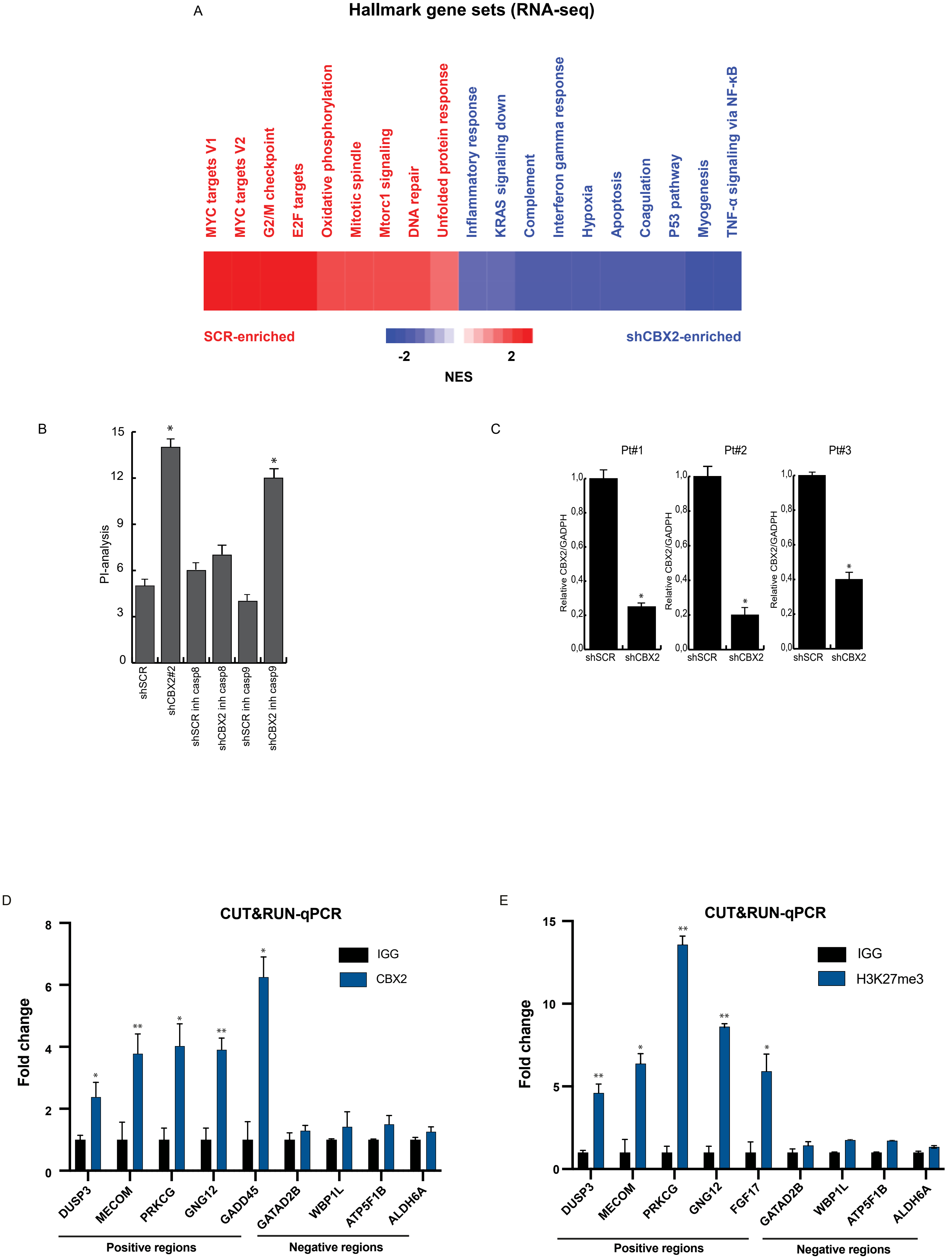

Supplementary figure 3

A

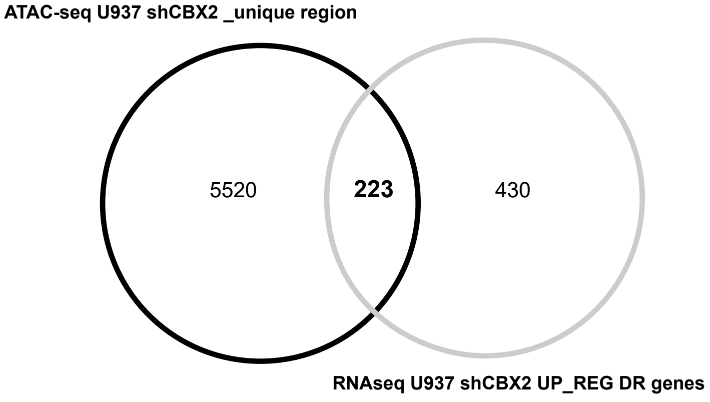

B

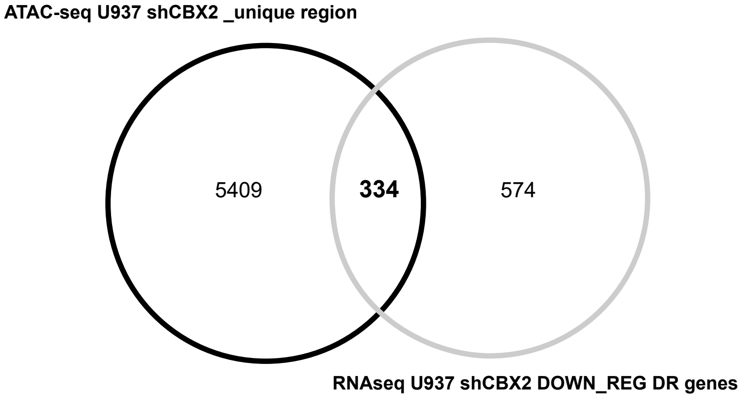

C

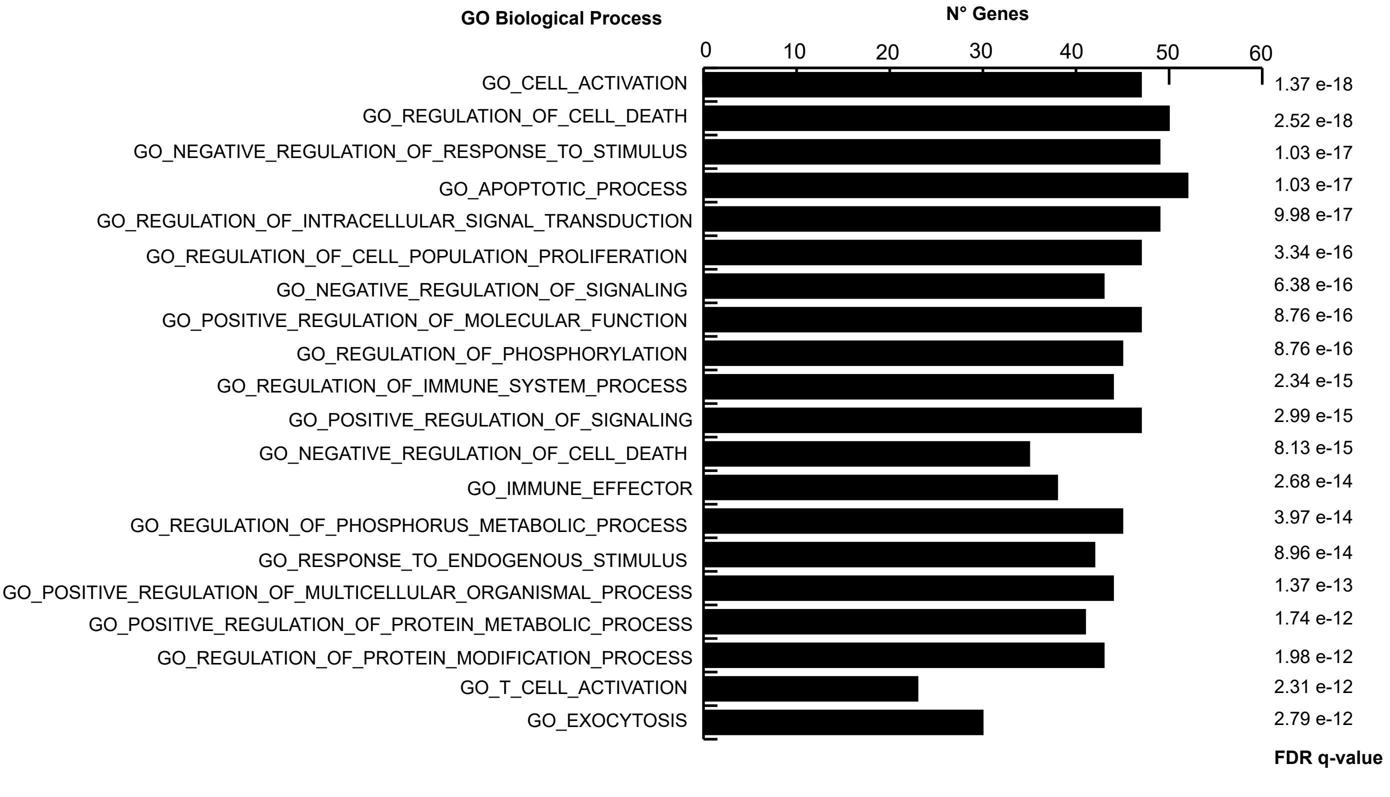

Supplementary figure 4

A

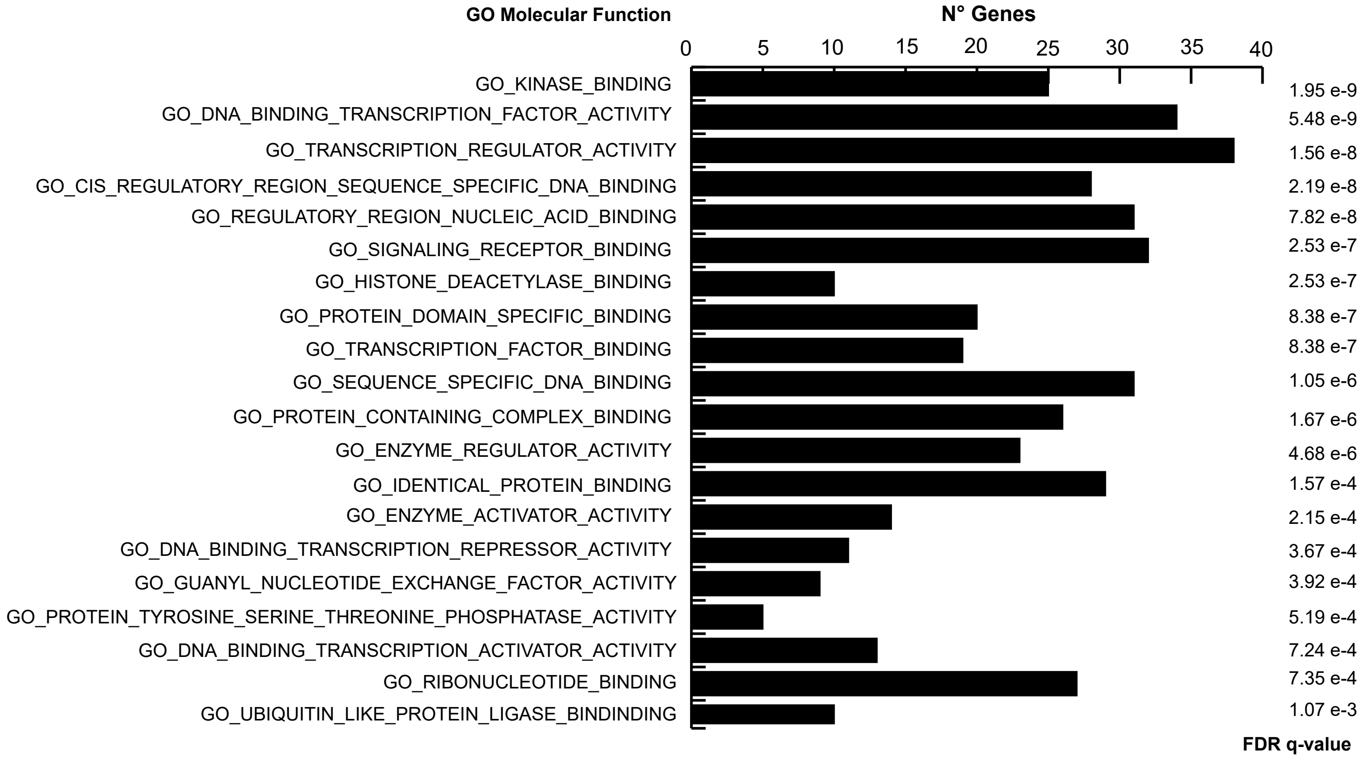

B

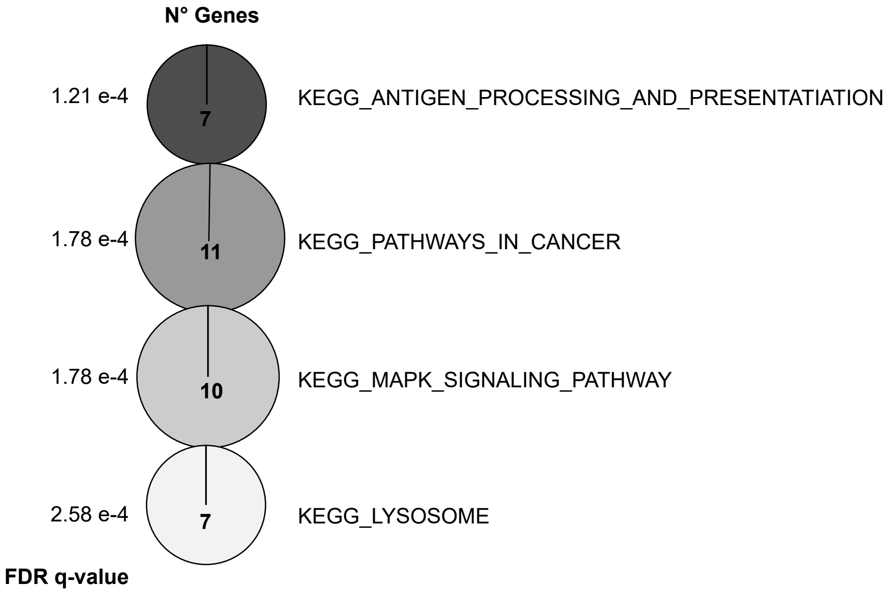

Supplement: Supplementary file 1 — Additional file 1: Supplementary Figure 1. CBX2 silencing impairs leukemic cell survival and induces cell death. A Western blot analysis of CBX2 protein levels in ex vivo leukemic blasts and CD34+ cells. Tubulin was used as loading control. Quantitative analysis (densitometry) of WB is shown as fold change value. B Bar plot representation of CBX2 expression in TGCA, blueprint and bloodspot datasets (P ≤ 0.01). C-F WB analysis showing silencing of CBX2 following shCBX2#1 and shCBX2#2 transduction in U937 (C), K562 (D), HL-60 (E), and NB4 (F) cells. ERK1/2 was used as loading control. G and L Proliferation assay with shCBX2(1), shCBX2(2), and shSCR in HL60 (G) and NB4 (L) cells; experiments were performed at 7 days of puromycin selection; error bars show SD of three biological replicates (*P < 0.05, **P ≤ 0.01). H and M CCK8 analysis of shCBX2- and shSCR-transduced HL60 (H) and NB4 (M) cells; experiments were performed at 7 days of puromycin selection; error bars show SD of three biological replicates (*P < 0.05, **P ≤ 0.01). I and N Percentage of PI-positive shCBX2- and shSCR-transduced HL60 (I) and NB4 (N) cells; experiments were performed at 7 days of puromycin selection; error bars show SD of three biological replicates (*P < 0.05, **P ≤ 0.01). Supplementary Figure 2. shCBX2 affects key pathways in AML. A GSEA analysis showing the top differential hallmark gene sets (nominal P < 0.05) associated with shSCR and shCBX2 cells (NES = normalized enrichment score). B Caspase activity assay of shCBX2- and shSCR-transduced U937 cells untreated or treated with 50 μM of caspase-8 or caspase-9 inhibitors; error bars show SD of three biological replicates. C RT-qPCR analysis showing CBX2 expression in three shCBX2#2- and shSCR-transduced primary AML samples (pt#1, pt#2 and pt#3). Experiments were performed at 3 days of puromycin selection; error bars show SD of two biological replicates (*P < 0.05, **P ≤ 0.01). D and E CUT&RUN-qPCR analysis showing enrichment of CBX2 (D) and [file 12943_2022_1603_MOESM1_ESM.pdf]
